# A summary of current NKG2D-based CAR clinical trials

**DOI:** 10.1093/immadv/ltab018

**Published:** 2021-08-13

**Authors:** Sophie Curio, Gustav Jonsson, Sonja Marinović

**Affiliations:** 1 Department of Life Sciences, Imperial College London, London, UK; 2 The University of Queensland Diamantina Institute, The University of Queensland, Woolloongabba, QLD 4102, Australia; 3 Institute of Molecular Biotechnology of the Austrian Academy of Sciences, Vienna, Austria; 4 Laboratory for Personalized Medicine, Division of Molecular Medicine, Ruder Boskovic Institute, Zagreb, Croatia; 5 Department of Histology and Embryology, Faculty of Medicine, University of Rijeka, Rijeka, Croatia

**Keywords:** NKG2D, CAR T cells, immunotherapy, adoptive cell transfer

## Abstract

Cancer immunotherapies have significantly improved patient survival and treatment options in recent years. Nonetheless, the success of immunotherapy is limited to certain cancer types and specific subgroups of patients, making the development of new therapeutic approaches a topic of ongoing research. Chimeric antigen receptor (CAR) cells are engineered immune cells that are programmed to specifically eliminate cancer cells. Ideally, a CAR recognizes antigens that are restricted to tumor cells to avoid off-target effects. NKG2D is an activating immunoreceptor and an important player in anti-tumor immunity due to its ability to recognize tumor cells and initiate an anti-tumor immune response. Ligands for NKG2D are expressed on malignant or stressed cells and typically absent from healthy tissue, making it a promising CAR candidate. Here, we provide a summary of past and ongoing NKG2D-based CAR clinical trials and comment on potential pitfalls.

Cancer is the second leading cause of death globally, exerting a tremendous financial burden on healthcare systems. Despite significant breakthroughs in diagnosis and treatment in recent decades, incidence and mortality rates continue to be high. Surgery, adjuvant chemotherapy, and radiotherapy remain the standard treatments to date, which, depending on the type of cancer often have low success rates. As a result, several therapeutic strategies are actively being developed, including chimeric antigen receptor (CAR)–T-cell–based immunotherapy, which is a promising therapeutic approach for cancer patients resistant to conventional therapies [1]. A CAR is a synthetic receptor allowing tumor-specific antigen recognition and consists of four main parts: (i) an extracellular recognition domain (ligand sensing), (ii) a hinge domain, (iii) a transmembrane domain, and (iv) an intracellular signaling domain [2]. The extracellular recognition domain of CAR constructs can consist of light and heavy variable chains from the single-chain variable fragment (scFv) from antibodies [3] or other engineered and artificial domains with strong affinities toward various ligands [4,5]. The intracellular signaling domain almost always consists of the CD3ζ signaling moiety. To enhance the downstream signaling strength of the CAR, the CD3 ζ signaling domain is often fused to one or more co-stimulatory domains [6].

NKG2D (natural-killer group 2, member D) is one of the best characterized activating immune receptors associated with tumor immunosurveillance. Both primary and metastatic cancer cells frequently upregulate stress-induced ligands that are closely related to MHC class I molecules such as MICA, MICB, and the ULBP family of proteins which are then recognized by the NKG2D receptor present on natural killer (NK) cells and various T cell subsets, including CD8^+^ and CD4^+^ αβ T cells and γδ T cells [7]. The interaction between the NKG2D ligand (NKG2DL) and the NKG2D receptor triggers the activation, expansion, and production of pro-inflammatory cytokines of effector immune cells, typically leading to target cell elimination [7] (Fig. 1A).

**Figure 1.**
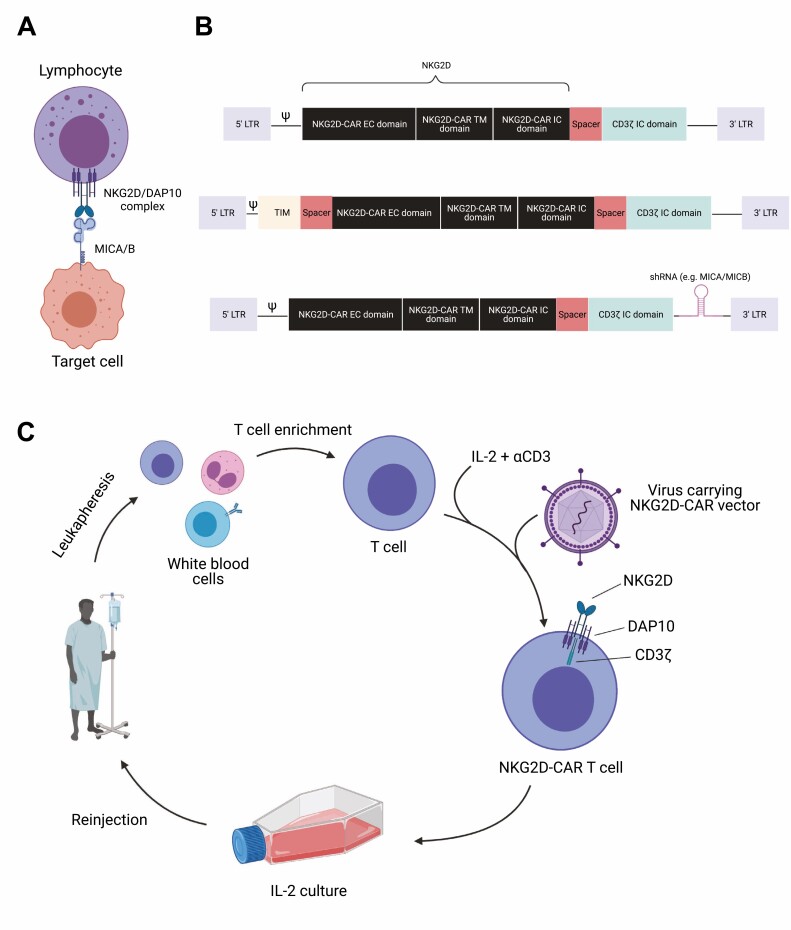
Overview of NKG2D-CAR T cell therapy. (A) Schematic of NKG2D on the surface of lymphocytes interacting with human ligands MICA and MICB. (B) Schematics of Celyad’s NKG2D-CAR constructs consisting of an extracellular and transmembrane NKG2D domain, a spacer and intracellular CD3 ς signaling domain. The base construct can also contain a TCR inhibitory molecule (TIM, middle) or shRNA targeting NKG2D ligands (bottom). (C) NKG2D-CAR T cell pipeline in the clinic. PBMCs are isolated from patients and enriched for T cells. Enriched T cells are expanded and transfected with the CAR, which is stabilized by endogenous DAP10, before being expanded and re-infused into the patient. Created using Biorender.com.

The role of NKG2D and its ligands in tumor immunosurveillance has been demonstrated in animal models, where it was shown that NKG2DLs confer an effective barrier to tumor formation and that NKG2D deficiency results in a decreased ability to fight tumor cells [8,9]. Findings of high NKG2DL expression in human colorectal, cervical, and nasopharyngeal carcinomas and its correlation with improved disease-free survival supported the role of NKG2D in anti-tumor immunity and the notion of NKG2D as a potential immunotherapy target [10]. In contrast to current FDA-approved CAR T cells, which are all based on the expression of a single-chain variable antibody fragment recognizing a tumor-specific antigen [11], NKG2D CAR T cells express an immunoreceptor, including its transmembrane signaling adaptors, that recognizes antigens expressed on the surface of tumor cells. Indeed, a number of clinical trials harnessing NKG2D as a CAR are currently ongoing, the majority of which are based on CAR T cells, with two trials using CAR NK cells and one trial using CAR γδT cells (Table 1). NK cells pose a few advantages over αβT cells: primarily, autologous transfer of NK cells has a lower degree of graft versus host disease (GvHD) and cytokine-release syndrome compared to αβT cells, which enables the possibility of engineering off-the-shelf CAR NK cell therapies [12]. γδT cells are tissue-resident immune cells with inherent anti-tumor function that do not rely on the expression of tumor-specific antigens and therefore provide a promising target for CAR cell therapy [13]. CAR γδT cells have been shown to have increased cytotoxicity compared to normal γδT cells and maintain their ability to cross-present antigens to conventional T cells [14]. Nonetheless, there are some potential pitfalls of CAR γδT cells and CAR NK cells. Preclinical data suggests that persistence of both cell types is limited and reduced compared to CAR αβT cells [15,16] and CAR NK cells can be sensitive to thawing and long-term storage [17]. Despite some of the apparent advantages, most importantly the possibility to develop off-the-shelf therapy, no CAR γδT cells or CAR NK cells have been approved to this date and more research is needed to determine whether they can perform as well, or better, than αβT cells.

**Table 1.** Summary of current clinical trials, including the treated condition and outcome (if applicable)

Trial number	Trial name	Drug name	Company/ University	NKG2D Construct/Treatment	Condition	Trial number	Outcome	Reference
NCT02203825		CM-CS1/ CYAD-01	Celyad Oncology	CAR T cell	AML, MM, MDS	NCT02203825	No objective clinical efficacy	[23]
							No significant CAR T cell expansion & persistence	[24]
							Multiple infusions are necessary	[18]
NCT03018405	THINK	CYAD-01		CAR T cell	5 solid cancers (CRC, ovarian, bladder, TNB, pancreatic), AML, MM	NCT03018405	Anti-leukemic activity in 6/13 patients	[33]
								[34]
NCT03692429	alloSHRINK	CYAD-101		CAR T cell (after standard chemotherapy)	Unresectable Metastatic CRC	NCT03692429	15 patients: 2 partial response, 9 stable disease	[35]
NCT03370198	LINK	CYAD-01		CAR T cell (hepatic transarterial infusion)	Unresectable CRC Liver Metastases	NCT03370198		[36]
NCT03310008	SHRINK	CYAD-01		CAR T cell + FOLFOX	CRC Liver Metastasis	NCT03310008		
NCT03466320	DEPLETHINK	CYAD-01		CAR T cell (after non-myeloablative preconditioning)	AML, MDS	NCT03466320		
NCT04167696	CYCLE-1	CYAD-02		CAR T cell (after non-myeloablative preconditioning)	AMD, MDS	NCT04167696		
NCT04107142	CTM-N2D-101	CTM-N2D	CytoMed Therapeutics Pte Ltd	CAR γδT cells	CRC, TNBC, Sarcoma, Nasopharyngeal Carcinoma, Prostate & Gastric Cancer	NCT04107142		
NCT04623944		NKX101	Nkarta Inc	CAR NK cell	AML, MDS, MDS	NCT04623944		[37]
NCT04717999		UWNKG2D	UWELL Biopharma	CAR T cell	Recurrent Glioblastoma	NCT04717999		
NCT03415100			The Third AffiliatedHospital of Guangzhou Medical University	CAR NK cells	Solid tumors	NCT03415100		
NCT04270461		KD-025	Jiujiang University Affiliated Hospital / KAEDI	CAR T cell	HCC, Glioblastoma, Medulloblastoma, CRC	NCT04270461	Successful in mice	[38]
NCT04550663		KD-025	The Affiliated Nanjing Drum Tower Hospital of Nanjing University Medical School / KAEDI	CAR T cell	Solid tumor, HCC, CRC, Glioma (relapsed or refractory)	NCT04550663	Successful in mice	[38]
NCT04658004			Zhejiang University / Yake Biotechnology Ltd.	CAR T cell	AML	NCT04658004		

AML, acute myeloid leukemia; CRC, colorectal cancer; HCC, hepatocellular carcinoma; MDS, myelodysplastic syndrome; MM, multiple myeloma; TNB, triple-negative breast cancer.

Celyad Oncology is the current frontrunner in terms of NKG2D-based CAR therapies. Their CAR construct, which is based on αβT cells, consists of extracellular, full-length human NKG2D (serving as both the extracellular recognition domain and transmembrane domain) fused to the intracellular signaling domain of CD3ς, which is stabilized through naturally expressed DAP10 (Fig. 1B top) [18,19]. Current versions of the NKG2D-CAR constructs can also contain TCR Inhibitory Molecule (TIM), a small peptide which is capable of interfering with endogenous TCR signaling thought to contribute GvHD (Fig. 1B middle) [20], or an short-hairpin RNA (shRNA) which targets NKG2D ligands MICA and MICB that can be naturally present on T cells, effectively preventing fratricide (Fig. 1B bottom) [21]. NKG2D-CAR T cells are generated in a multistep process. First, white blood cells are isolated from patients through leukapheresis and enriched for T cells. Enriched T cells are activated using IL-2 and αCD3, and the CAR construct transduced using an SFG retroviral vector [22]. These NKG2D-CAR T cells are then expanded in media containing IL-2 before being re-injected into the patients (Fig. 1C) [18,19].

Celyad Oncology is currently testing three different CAR T cells in various types of cancer. CYAD-01 (formerly known as NKR-2) is an autologous NKG2D-CAR T cell, which is being tested alone or in combination with chemotherapy in hematological and solid cancers. An improved version of CYAD-01—CYAD-02—which, in addition to the NKG2D receptor, contains the shRNA that targets and knocks down expression of NKG2D ligands on CAR T cells (Fig. 1a bottom), is being trialed in acute myeloid leukemia (AML) and myelodysplastic syndrome (MDS) patients [18,23,24] (Table 1). In addition to the autologous therapy, Celyad Oncology has developed an allogeneic NKG2D-CAR T cell—CYAD-101—for use in patients suffering from unresectable metastatic colorectal cancer (Fig. 2). CYAD-01 was initially tested as a single infusion (NCT02203825), which resulted in no clinical efficacy (Fig. 2 and Table 1). Following this initial trial, four further trials were initiated—THINK (NCT03018405), a dose escalation trial; SHRINK (NCT03310008) a dose-escalation trial administered concurrently with chemotherapy (FOLFOX) in patients with colon cancer liver metastasis; LINK (NCT03370198), a dose-escalation trial with hepatic transarterial infusion, as opposed to i.v. infusion and DEPLETHINK (NCT03466320) a dose-escalation trial with infusion following CyFlu preconditioning in AML and MDS patients. Following the SHRINK trial, the allogeneic NKG2D-CAR T cell was tested in the alloSHRINK trial (NCT03692429), in which CYAD-101 was administered following standard chemotherapy. Celyad Oncology recently announced a collaboration with Merck to test the combination of KEYTRUDA® (pembrolizumab, anti PD-1 antibody) and CYAD-101 in the KEYNOTE-B79 trial, in which they hope to enhance the function of CYAD-101 in the tumor microenvironment and induce a more durable anti-tumor response. Similar to tumor-infiltrating T cells, CAR T cells can upregulate PD-1, impairing their function, and PD-1 blockade might therefore improve CAR T cell efficacy and function [25]. The CYCLE-1 trial (NCT04167696), which was initiated in 2019, is seeking out to test the safety and efficacy of CYAD-02 following CyFlu preconditioning in AML and MDS patients (Fig. 2 and Table 1).

**Figure 2.**
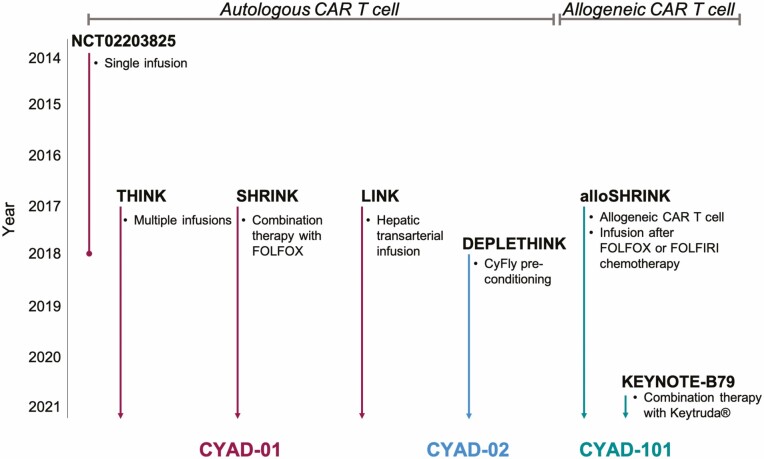
Overview and timeline of clinical trials testing the three different NKG2D-CAR T cells developed by Celyad Oncology. CYAD-01, CYAD-02, and CYAD-101 were tested in various clinical trials with different infusion strategies, combination therapies or preconditioning.

While preliminary results are promising, there are some potential pitfalls to be considered. For one, the presence of NKG2D ligands is not always associated with an enhanced cytolytic immune response against cancer. NKG2D-mediated tumor immune surveillance can exert considerable selection pressure on tumor survival [26]. Therefore, it is not surprising that some human tumors shed NKG2DL from their surface to evade the immune response and therefore produce high levels of a soluble version of NKG2DL. Binding of soluble NKG2D ligand can then result in systemic desensitization of NKG2D in effector cells and impaired anti-tumor function [27–29]. Therefore, although the NKG2D receptor may represent a promising treatment strategy for cancer therapy, it should be considered that high levels of soluble NKG2DL may impair immune cell responsiveness by downregulating the NKG2D receptor. Indeed, strategies have been developed to prevent shedding of NKG2DL from the surface of tumor cells [30], which if combined with NKG2D CAR T cell therapy could increase the anti-tumor activity and overcome tumor immune evasion. In addition to ligand shedding and immune evasion, another major concern is that NKG2DL, which is typically absent from healthy tissue, is constitutively expressed on healthy intestinal epithelial cells [31], potentially leading to off-target effects in NKG2D-based immunotherapy. Further, NKG2D has been shown to contribute to tumorigenesis in the setting of inflammation-driven cancer, such as a model of hepatocellular carcinoma [32] and whether NKG2D CAR T cells would contribute to the anti-tumor function or instead foster the tumor-promoting inflammation remains to be determined.

## Outlook

The therapeutic potential of NKG2D CAR T cells is high due to the ubiquitous expression of NKGDL on various cancers and the well-studied anti-tumor function of NKG2D-expressing immune cells. Preliminary data from clinical trials testing NKG2D CAR T cells are promising and research developing improved CAR constructs or combination therapy are ongoing. While most trials utilize αβT cells, NKG2D CAR NK cells or γδ CAR T cells encompass a novel approach that will allow autologous cell transfer and therefore harbors the potential for off-the-shelf therapies.

## Data Availability

Not applicable.

## Abbreviations

AML: Acute myeloid leukemia
CAR: Chimeric antigen receptor
MDS: Myelodysplastic syndrome
NK: Natural killer
NKG2D: Natural-killer group 2, member D
NKG2DL: NKG2D ligand
scFV: Single-chain variable fragment
shRNA: Short-hairpin RNA
TIM: TCR inhibitory molecule
